# Efficacy of an exoskeleton-based physical therapy program for non-ambulatory patients during subacute stroke rehabilitation: a randomized controlled trial

**DOI:** 10.1186/s12984-021-00942-z

**Published:** 2021-10-10

**Authors:** Dennis R. Louie, W. Ben Mortenson, Melanie Durocher, Amy Schneeberg, Robert Teasell, Jennifer Yao, Janice J. Eng

**Affiliations:** 1 Department of Physical Therapy, University of British Columbia, 212-2177 Wesbrook Mall, Vancouver, BC V6T 1Z3 Canada; 2 Rehabilitation Research Program, Vancouver Coastal Health Research Institute, Vancouver, BC Canada; 3 Department of Occupational Science and Occupational Therapy, University of British Columbia, Vancouver, BC Canada; 4 Glenrose Rehabilitation Hospital, Alberta Health Services, Edmonton, AB Canada; 5 Parkwood Institute, St. Joseph’s Health Care, London, ON Canada; 6 Department of Physical Medicine and Rehabilitation, Schulich School of Medicine and Dentistry, Western University, London, ON Canada; 7 GF Strong Rehabilitation Centre, Vancouver Coastal Health, Vancouver, BC Canada; 8 Division of Physical Medicine and Rehabilitation, Faculty of Medicine, University of British Columbia, Vancouver, BC Canada

**Keywords:** Stroke, Rehabilitation, Walking, Exoskeleton, Physical therapy techniques, Clinical trial

## Abstract

**Background:**

Individuals requiring greater physical assistance to practice walking complete fewer steps in physical therapy during subacute stroke rehabilitation. Powered exoskeletons have been developed to allow repetitious overground gait training for individuals with lower limb weakness. The objective of this study was to determine the efficacy of exoskeleton-based physical therapy training during subacute rehabilitation for walking recovery in non-ambulatory patients with stroke.

**Methods:**

An assessor-blinded randomized controlled trial was conducted at 3 inpatient rehabilitation hospitals. Patients with subacute stroke (< 3 months) who were unable to walk without substantial assistance (Functional Ambulation Category rating of 0 or 1) were randomly assigned to receive exoskeleton-based or standard physical therapy during rehabilitation, until discharge or a maximum of 8 weeks. The experimental protocol replaced 75% of standard physical therapy sessions with individualized exoskeleton-based sessions to increase standing and stepping repetition, with the possibility of weaning off the device. The primary outcome was walking ability, measured using the Functional Ambulation Category. Secondary outcomes were gait speed, distance walked on the 6-Minute Walk Test, days to achieve unassisted gait, lower extremity motor function (Fugl-Meyer Assessment), Berg Balance Scale, Patient Health Questionnaire, Montreal Cognitive Assessment, and 36-Item Short Form Survey, measured post-intervention and after 6 months.

**Results:**

Thirty-six patients with stroke (mean 39 days post-stroke) were randomized (Exoskeleton = 19, Usual Care = 17). On intention-to-treat analysis, no significant between-group differences were found in the primary or secondary outcomes at post-intervention or after 6 months. Five participants randomized to the Exoskeleton group did not receive the protocol as planned and thus exploratory as-treated and per-protocol analyses were undertaken. The as-treated analysis found that those adhering to exoskeleton-based physical therapy regained independent walking earlier (p = 0.03) and had greater gait speed (p = 0.04) and 6MWT (p = 0.03) at 6 months; however, these differences were not significant in the per-protocol analysis. No serious adverse events were reported.

**Conclusions:**

This study found that exoskeleton-based physical therapy does not result in greater improvements in walking independence than standard care but can be safely administered at no detriment to patient outcomes.

*Clinical Trial Registration* The Exoskeleton for post-Stroke Recovery of Ambulation (ExStRA) trial was registered at ClinicalTrials.gov (NCT02995265, first registered: December 16, 2016)

## Background

Recovering the ability to walk is commonly cited by patients with stroke as a top priority of both rehabilitation and research efforts [1, 2]. Besides predicting long-term mobility and community reintegration after stroke [3, 4], walking outcomes are also associated with cognitive performance, post-stroke depression, and quality of life [5–7]. With implications for so many post-stroke outcomes, refining rehabilitation efforts to optimize the timeliness and degree of walking recovery after stroke remains a top priority.

It is recommended that early stroke rehabilitation should be goal-oriented, repetitive, progressive, and task-specific to take advantage of neuroplastic recovery and make gains in mobility and walking [8, 9]. However, dependent patients requiring substantial assistance from one or two therapists are the least likely to achieve these guidelines and take very few steps during stroke rehabilitation; studies observing inpatient stroke rehabilitation have reported as low as 8 minutes of physical activity and progression from 6 to only 16 completed steps during physical therapy [10, 11]. Even with the introduction of body weight-supported treadmill training in stroke rehabilitation and promising findings for walking recovery, the physical demand of having multiple therapists involved to assist moving the lower extremities has limited its clinical application [12, 13]. With such minimal levels of walking practice, it is unsurprising that nearly half of patients admitted for stroke are discharged from rehabilitation without the ability to walk independently [14], which in turn influences meaningful outcomes such as discharge location and return-to-work [4, 15]. Consequently, it is those patients who are more impaired and unable to walk independently who should be the target of novel interventions and research [16]. Repetition and progression should be included as key components of such efforts.

Powered exoskeletons have been commercially developed to assist and automate overground walking for individuals with lower extremity weakness. Such devices strap around the lower limbs and generate joint motion using embedded motors. They may allow patients to achieve the higher duration and repetition of stepping practice recommended for stroke rehabilitation, while offloading therapists’ physical burden. However, previous research of treadmill-based robotic devices [e.g., Lokomat (Hocoma, Zurich, Switzerland)] has found mixed results for gait recovery; several randomized trials did not did not find superior effects of robotic training on walking outcomes [17, 18], yet several reviews have found improved walking independence [19, 20]. Powered exoskeletons may offer more realistic task-specific and goal-oriented overground walking practice than treadmill-based devices, as they address the criticism that suspended robotic devices lack variability in movement and encourage passive participation [16]. Early research of powered exoskeletons in stroke rehabilitation has shown promising findings, though only a few randomized controlled studies have been conducted and none have focused explicitly on non-ambulatory patients during the subacute phase of recovery [21]. From the previous reviews of electromechanically-assisted gait training, it has been recommended that further research and therapy with robotics should only be used with patients in the early phase of stroke recovery and who require more physical assistance to walk [16, 20, 22].

Although early research of powered exoskeletons has shown they can be safely used as an adjunct therapy [21, 23], limited research has investigated their effect when integrated within the standard physical therapy component of subacute stroke rehabilitation. The primary objective of this study was to assess the effect of an exoskeleton-based physical therapy program on the recovery of walking ability during subacute stroke rehabilitation. The primary hypothesis was that non-ambulatory patients who regularly utilized an exoskeleton during their physical therapy sessions would have greater walking independence at discharge compared to patients who received standard physical therapy. The secondary objective was to evaluate the effect of exoskeleton-based physical therapy on additional walking and mobility outcomes (e.g., speed), leg motor impairment, balance, cognition, post-stroke depression, and quality of life, at discharge and after 6 months.

## Methods

The full protocol and design of this multicenter, parallel-group, randomized controlled trial have been described elsewhere [24]. Approval was granted by each respective local research ethics board and operational institute to conduct the study in Vancouver, Edmonton, and London, Canada. The study and intervention are reported using the Consolidated Standards of Reporting Trials (CONSORT) and Template for Intervention Description and Replication guidelines (TIDieR) [25, 26].

### Participants

Participants were recruited from the following three inpatient rehabilitation hospitals: GF Strong Rehabilitation Centre (May 2017–March 2020), Glenrose Rehabilitation Hospital (December 2017–August 2019), and Parkwood Institute (November 2018–March 2020). Inclusion criteria for the study were: age of 19 years or older; stroke within the last 12 weeks (ischemic or hemorrhagic); one-sided hemiparesis; requiring significant assistance from one or two therapists to walk (Functional Ambulation Category [27] rating of 0 or 1); ability to understand and follow directions in English; ability to communicate (yes/no verbal or physical indication); and scheduled to receive physical therapy. Individuals were excluded if they had: a significant musculoskeletal or other neurological condition affecting mobility; or co-morbidities that would preclude activity (e.g., cardiovascular contraindications, pain which was intolerably worsened with exercise). Participants were also excluded if they were unable to walk prior to their stroke or had any contraindications to using the exoskeleton (e.g., pregnancy, leg length discrepancy, height/weight restrictions, open ulcerations at device contact points, etc.).

Following baseline testing, participants were randomized and allocated at a one-to-one ratio to either the Exoskeleton group or Usual Care group using a third-party, online randomization service (www.randomize.net, Interrand Inc., Ottawa, ON) which generated and concealed the allocation sequence. A permuted block design (block sizes: 2, 4) was stratified by site to control for potential differences in standard of care, and by physical function using the Berg Balance Scale (score of < 12 or ≥ 12, which is the threshold that predicts a non-ambulator to regain unassisted ambulation [28]). The research coordinator at each site conducted the randomization after the baseline assessment.

### Exoskeleton device

This study utilized the EksoGT powered exoskeleton (Ekso Bionics, Richmond, California, USA). This exoskeleton straps bilaterally to the lower extremities, and has electrically actuated hip and knee joints, and a passive spring-loaded ankle articulation which supports toe-off and foot clearance via a footplate. The EksoGT software can be programmed by the operating therapist to power the user’s lower limbs in a walking pattern, providing partial or complete assistance. Other training considerations, including gait parameters (e.g., step height, length, swing speed) and walking automaticity (i.e., how each step is triggered), can also be programmed to tailor the gait training and challenge the user according to their ability. The assistance provided to each leg can be programmed separately, further allowing clinicians to individualize gait training. The device software reports standing and walking time, as well as step counts, per use. The device manufacturer did not play any role (design, conduct, reporting) in this research study.

### Interventions

Individualized exoskeleton-based gait training was provided to participants in the Exoskeleton group during their standard weekly physical therapy sessions. Physical therapists were given the choice to replace 75% of their weekly sessions entirely with exoskeleton training or to perform exoskeleton training each session for 75% of therapy time. Therapists unanimously opted to replace full physical therapy sessions with the exoskeleton training; because physical therapy standard of care varied between sites (4–5 days a week, for 45–60 min per session), physical therapists were thus negotiated to provide 60-min exoskeleton-based sessions, 3 times a week. During exoskeleton intervention sessions, participants wore the device and were guided to achieve as much repetitious stepping and walking practice as possible. Hospital therapists who were certified to use the exoskeleton (by the manufacturer) carried out the intervention, progressing the training to reduce the level of assistance provided by the device and to increase the amount of time spent walking. With previous evidence showing that robotics-assisted gait training is no more effective than overground walking for ambulatory patients with stroke [20, 29], therapists had the option to discontinue use of the exoskeleton once the participant achieved a threshold level of independence in walking. However, they were instructed to continue focusing on gait training for 75% of their weekly physical therapy sessions if the exoskeleton was discontinued. Guidelines for adapting and progressing gait training using the exoskeleton device, suggested training duration and step count targets (≥ 25 min of walking and ≥ 700 steps per session by the fourth week of exoskeletal gait training), as well as an algorithm to assist decision-making to discontinue use of the exoskeleton, were provided to intervention therapists [24]. The remaining 25% of weekly physical therapy sessions allowed the therapists to work on other goals of their choice (e.g., discharge planning, upper extremity, pain management). Therapists monitored participants for adverse events before, during, and after each training session.

Usual Care participants received standard physical therapy care throughout their rehabilitation stay. Though standard of care varied between sites, patients typically received physical therapy 4–5 days a week, for 45–60 min per session. Therapists providing Usual Care were not provided specific instructions or limitations, other than avoiding use of the robotic exoskeleton. Physical therapy during stroke rehabilitation is typically provided with patient-specific goals in mind and typically focuses on mobility and gait training.

The respective interventions were delivered to both groups until discharge, to a maximum of 8 weeks; this 8-week maximum duration was selected to reflect recommended and actual rehabilitation stay [8, 30]. Time spent physically upright (standing or walking, regardless of assistance) and step count during physical therapy sessions were monitored twice a week using the activPAL3 micro (PAL Technologies, Glasgow, UK) activity tracker to estimate and compare the amount of daily mobility practice provided to each group. The activPAL system provides valid and accurate measures of physical activity and step counts in the inpatient hospital setting [31, 32]. Participants in either group who were not yet discharged by 8 weeks received standard physical therapy care (without any exoskeleton use) beyond the intervention period, at the discretion of their care team.

### Outcome measures

Participants were assessed at baseline (before randomization), at discharge (or after 8 weeks of the intervention), and at 6-month follow-up (relative to study enrollment) by a blinded assessor. Additional demographic data to describe the sample were collected prior to randomization, including age, sex, time since stroke, and stroke characteristics (side, type, recurrence, severity).

The primary outcome was walking ability at discharge, measured using the Functional Ambulation Category (FAC) [27]. The FAC is a 6-item ordinal scale that classifies the level of support needed to walk safely, irrespective of the use of a lower extremity orthosis or walking aid, ranging from 0 (unable to walk without the assistance of two people) to 5 (independent walking on uneven surfaces and on stairs). The FAC has good test–retest reliability and validity in the post-stroke population and is responsive to change in the subacute phase of stroke [33]; additionally, it can still be scored for individuals who are unable to walk independently. By definition of its values, each gradation of the FAC is an inherently clinically important difference. Walking ability was measured again at 6 months as a secondary outcome.

Other secondary outcomes at discharge and 6 months included additional measures of walking and mobility, motor function, balance, mood, cognition, and quality of life. Additional walking outcomes were gait speed during a 5-meter walk test [34, 35] and distance walked during the 6 Minute Walk Test (6MWT) [36, 37], measured only at discharge and 6-month follow-up if the participant could complete the task without manual assistance (FAC ≥ 3). The number of days during the intervention period to achieve unassisted ambulation (FAC ≥ 3) was also recorded, monitored weekly through communication with the intervention therapists. Motor function of the leg was assessed using the lower extremity subscale of the Fugl-Meyer Assessment (FMA-LE, score range 0–34) [38, 39], with higher scores indicating better motor function. Balance was assessed using the Berg Balance Scale (score range 0–56) [40, 41], with higher scores indicating better balance. Mood was assessed using the Patient Health Questionnaire (score range 0–27) [42, 43], with higher scores indicating greater presence of depressive symptoms. Cognition was assessed using the Montreal Cognitive Assessment (MoCA, score range 0–30) [44, 45], with lower scores indicating cognitive impairment. Quality of life was assessed using the Medical Outcomes Short-Form 36, which is a multi-purpose health survey comprised of 36 questions on functional health and well-being [46]. The items can be aggregated and standardised to provide a physical and mental health component summary score, with higher scores indicating better health-related quality of life [47].

### Statistical analysis

Data were analyzed using RStudio (Version 1.3.959) (RStudio, Boston, MA, USA) running on R (Version 4.0.1) (R Foundation for Statistical Computing, Vienna, Austria). Descriptive statistics were expressed as mean (standard deviation, SD) for continuous variables, median (interquartile range, IQR) for ordinal variables, and counts (percentages) for categorical variables. Following the intention-to-treat principle, all participants were analyzed according to their original treatment allocation. For the primary outcome (FAC), between-group differences in change scores from baseline were analyzed using the Mann–Whitney *U* test at discharge and 6-month follow-up. Between-group differences for secondary walking outcomes were analyzed at discharge and 6-month follow-up using independent *t*-test for continuous variables and Mann–Whitney *U* test for ordinal or non-normally distributed continuous variables (Shapiro–Wilk test *p* < 0.05). A gait speed of 0 m/s and 6MWT distance of 0 m were appended for those who were unable to complete the respective measures (i.e., had not achieved FAC ≥ 3) at discharge and at 6-month follow-up. Differences in secondary outcomes of impairment, balance, cognition, mood, and quality of life were examined using analysis of covariance (ANCOVA), using baseline score as covariate.

We addressed two missing data points at post-intervention by carrying forward baseline observation, while last observation carried forward was used for missing data points at 6 months [48]. A sensitivity analysis was performed on the missing data, comparing ± 25% of the last value carried forward, for any intention-to-treat comparison with significant findings.

We also performed exploratory as-treated and per-protocol analyses, as some of the participants in the intervention group declined further use of the exoskeleton and instead received standard physical therapy until their discharge assessment [49]. For the as-treated analysis, participants were analyzed according to the intervention they actually received. Thus, Exoskeleton group participants who underwent less than 70% of possible exoskeleton sessions for the time they were in the trial were analyzed as part of the Usual Care group. For the per-protocol analysis, wherein only those participants who received their allocated intervention are analyzed, those same participants who underwent less than 70% of possible exoskeleton sessions were removed from the total study sample altogether.

Sample size was calculated a priori, based on the experimental group achieving a 2-point difference in improvement on the FAC compared with the control group. The calculation was also based on an estimated SD of 2.0, derived from a previous study examining the FAC in subacute stroke [33]. Using a significance threshold of 0.05 and power set to 80%, a sample size of 16 participants in each group was required. To account for 20% drop-out and loss to follow-up, a sample size of 20 participants in each group was targeted.

## Results

**Fig. 1:**
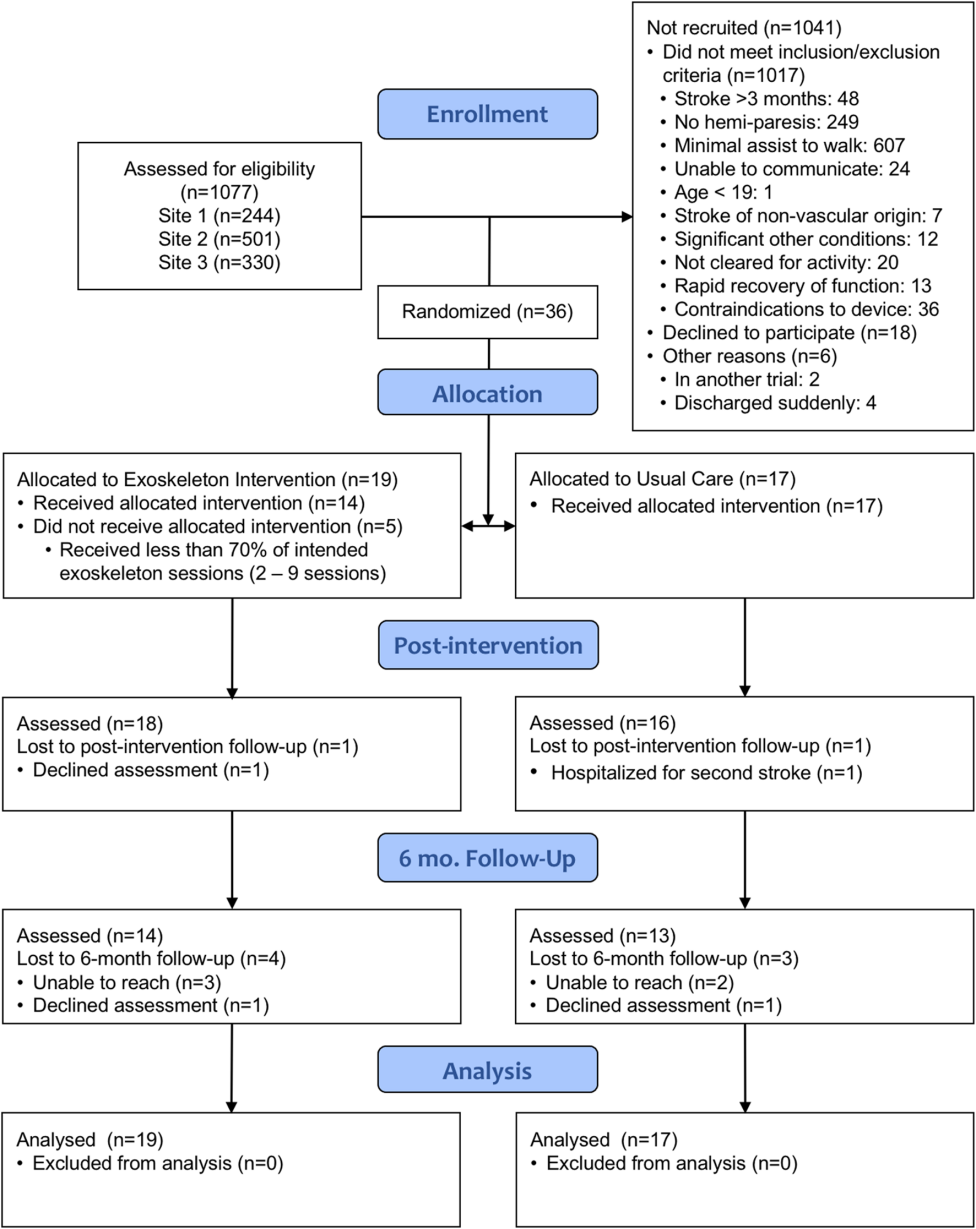
Flow diagram of study participants

Thirty-six participants were recruited and randomized between 5 May 2017 and 9 March 2020. Due to a suspension of research activities as a result of COVID-19, the trial was terminated early. The flow of participants through the trial is displayed in Fig. 1. Five participants dropped out from the Exoskeleton group and received standard physical therapy for the remainder of the intervention period. Of these, three participants reported simply not liking the device and did not wish to continue the training. One participant reported knee pain which persisted only while using the device, which could not be resolved through sizing or kinematic adjustments. Another participant reported severe fatigue as their reason for discontinuing the exoskeleton. All but one participant in each group was assessed for the discharge evaluation. Seven additional participants were lost to follow-up for the 6-month evaluation, either declining or were unable to be reached.

Table 1 lists the demographic characteristics of all study participants. Fewer females participated in the study (10 females vs 26 males), and there was a lower proportion of females randomized to the Exoskeleton group than the Usual Care group [3 (15.8%) vs. 7 (41.2%)]. There were more participants with hemorrhagic stroke in the Exoskeleton group than the Usual Care group [7 (36.8%) vs. 4 (23.5%)], but fewer participants hospitalized for recurrent stroke [4 (21.1%) vs. 7 (41.2%)].

**Table 1:** Demographic characteristics

	Exoskeleton group n = 19	Usual Care group n = 17
Age, in years, mean (SD)	59.6 (15.8)	55.3 (10.6)
Sex, male, n (%)	16 (84)	10 (59)
Days since stroke, mean (SD)	36.7 (19.0)	40.9 (19.8)
Side of paresis, left, n (%)	11 (58)	10 (59)
Type of stroke		
Ischemic, n (%)	12 (63)	13 (76)
Hemorrhagic, n (%)	7 (37)	4 (24)
Recurrent stroke, yes, n (%)	4 (21)	7 (41)

*NIHSS* National Institute of Health Stroke Scale, *SD* standard deviation

The trial intervention period lasted a mean (SD) of 48 (11) days for participants in the Exoskeleton group and 50 (11) days for the Usual Care group. A total of 12 participants reached the 8-week maximum intervention duration, of which 6 were in the Exoskeleton group. Participants in the Exoskeleton group underwent a mean (SD) of 11 (5) exoskeleton training sessions in the first 27 (16) days in the trial before fully discontinuing the device, at 2.9 (0.4) sessions per week. Exoskeleton participants performed a mean (SD) of 592 (332) steps per physical therapy session, while Usual Care participants performed 330 (355) steps per session. Exoskeleton participants were physically upright for a mean (SD) of 33.4 (7.6) min per intervention session, compared to 21.8 (6.0) min for the Usual Care group.

Apart from the above-mentioned reasons for dropouts from the Exoskeleton group, no other notable adverse events relating to the exoskeleton were reported. Three additional participants experienced transient pain or discomfort while using the exoskeleton, which did not affect their intervention adherence, that was easily resolved within the session through device sizing adjustments. One participant in the Usual Care group experienced a second stroke at the end of their rehabilitation stay and was re-admitted to acute care.

In the intention-to-treat analysis of the primary outcome, there were no statistically significant differences between groups in change score from baseline in the FAC at either discharge or 6 months. Table 2 shows the median FAC score for each group at baseline, discharge, and 6 months, as well as change from baseline scores. For the secondary walking outcomes, there were no significant between-group differences at discharge or at 6 months (Table 3). A total of 26 participants (Exoskeleton: 12, Usual Care: 14) became ambulatory without requiring physical assistance (FAC ≥ 3) during the intervention period; for these participants, there was no difference in the time to achieve unassisted ambulation between groups.

**Table 2:** Primary outcome analysis

FAC	Exoskeleton n = 19 Median (IQR)	Usual Care n = 17 Median (IQR)	*p*-value
Baseline	0 (0–1)	0 (0–1)	
Discharge	3 (1–4)	3 (3–4)	
Change from baseline	2 (1–4)	3 (2–3)	0.72^a^
6-month	4 (2–5)	4 (3–5)	
Change from baseline	3 (2–4)	3 (3–4)	0.65^a^

*FAC* Functional Ambulation Category, *IQR* interquartile range

^a^Analyzed using Mann–Whitney U test

**Table 3:** Secondary walking outcomes

Variable	Exoskeleton n = 19 Mean (SD)	Usual Care n = 17 Mean (SD)	*p*-value
Gait speed, m/s			
Discharge	0.38 (0.3)	0.35 (0.3)	0.99^a^
6-month follow-up	0.52 (0.5)	0.42 (0.3)	0.74^a^
6MWT, m			
Discharge	117.0 (112.7)	93.0 (84.0)	0.72^a^
6-month follow-up	164.5 (152.8)	123.4 (90.1)	0.60^a^
Days to unassisted walking^b^	26.8 (13.3)	35.3 (15.7)	0.16^c^

*6MWT* 6-minute walk test

^a^Analyzed using Mann–Whitney U test

^b^Exoskeleton n = 12, Usual Care n = 14

^c^Analyzed using independent *t*-test

Secondary outcomes of impairment, balance, mood, cognition, and quality of life at all timepoints are summarized in Table 4. After adjusting for baseline score, no significant group effects were found at either discharge or 6-month follow-up. Sensitivity analyses were not performed given the lack of significant findings.

**Table 4:** Secondary outcomes of impairment, balance, mood, cognition, and quality of life

Variable	Exoskeleton n = 19 Mean (SD)	Usual Care n = 17 Mean (SD)	Group difference (95% CI)	F-statistic	*p*-value^a^
FMA-lower					
Baseline	17.3 (6.6)	17.5 (7.0)			
Discharge	23.0 (5.9)	20.8 (7.1)	2.3 (− 0.4–5.1)	F(1,33) = 2.95	0.09
6-month	23.5 (6.0)	22.0 (5.2)	1.6 (− 1.4–4.5)	F(1,33) = 1.19	0.28
BBS					
Baseline	15.3 (10.0)	19.2 (15.4)			
Discharge	36.6 (15.1)	37.8 (17.3)	1.4 (− 8.2–10.9)	F(1,33) = 0.086	0.77
6-month	40.3 (14.3)	43.0 (15.6)	− 0.5 (− 9.0–7.9)	F(1,33) = 0.017	0.90
PHQ-9					
Baseline	7.2 (4.2)	7.7 (6.4)			
Discharge	4.1 (3.3)	6.1 (7.4)	− 1.6 (− 4.5–1.3)	F(1,33) = 1.289	0.26
6-month	5.1 (4.0)	6.8 (6.5)	− 1.4 (− 3.6–0.8)	F(1,33) = 1.599	0.22
MoCA^b^					
Baseline	22.4 (4.3)	23.5 (5.0)			
Discharge	24.9 (5.2)	24.6 (5.2)	1.5 (− 0.2–3.1)	F(1,29) = 3.456	0.07
6-month	24.6 (4.8)	25.1 (4.9)	0.4 (− 1.6–2.4)	F(1,29) = 0.185	0.67
SF-36-physical^c^					
Baseline	30.2 (8.9)	28.2 (6.5)			
Discharge	31.8 (9.7)	28.7 (8.8)	2.3 (− 3.9–8.5)	F(1,31) = 0.591	0.45
6-month	33.5 (9.9)	30.8 (10.5)	1.9 (− 5.1–8.8)	F(1,31) = 0.298	0.59
SF-36-mental^c^					
Baseline	51.0 (10.4)	49.4 (12.4)			
Discharge	52.5 (12.6)	52.6 (14.7)	− 1.1 (− 9.3–7.0)	F(1,31) = 0.079	0.78
6-month	50.1 (12.5)	52.4 (13.2)	− 3.2 (− 11.0–4.5)	F(1,31) = 0.723	0.40

*BBS* Berg Balance Scale, *FMA-LE* lower extremity component of Fugl-Meyer Assessment, *MoCA* Montreal cognitive assessment, *PHQ-9* Patient Health Questionnaire, *SD* standard deviation, *SF-36-Mental* mental component of 36-item short form survey, *SF-36-Physical* physical component of 36-item short form survey

^a^All outcomes analyzed using ANCOVA, using baseline score as covariate

^b^Exoskeleton n = 17; Usual Care n = 15

^c^Exoskeleton n = 18; Usual Care n = 16

Findings from the as-treated and per-protocol analyses of primary and secondary walking outcomes are presented in Table 5. In the as-treated analysis, participants adhering to the Exoskeleton protocol who achieved unassisted ambulation did so significantly earlier in the intervention period than Usual Care participants who became ambulatory (*p* = 0.03). While not different at discharge, there was a significant difference between groups at 6 months for both gait speed (*p* = 0.04) and 6MWT (*p* = 0.03). However, these differences between groups were not significant in the per-protocol analysis. For other secondary outcomes, there was a significant effect of exoskeleton training on FMA-LE [as-treated adjusted group difference: 3.9, 95% CI 1.3–6.6, F(1,33) = 9.33, *p* = 0.004; per-protocol adjusted group difference: 3.7, 95% CI 0.9–6.5, F(1,28) = 7.29, *p* = 0.01] and MoCA [as-treated adjusted group difference: 2.1, 95% CI 0.6–3.7, F(1,29) = 7.96, *p* = 0.009; per-protocol adjusted group difference: 2.0, 95% CI 0.4–3.6, F(1,25) = 6.62, *p* = 0.02] at discharge, though this did not carry over at the 6-month evaluation.

**Table 5:** As-treated and per-protocol analyses of primary and secondary walking outcomes

Variable	As-treated			Per-protocol		
	Exoskeleton n = 14	Usual Care n = 22	*p*-value	Exoskeleton n = 14	Usual Care n = 17	*p*-value
FAC, median (IQR)						
Baseline	0 (0–1)	0 (0–1)		0 (0–1)	0 (0–1)	
Discharge	4 (3–4)	3 (2–4)		4 (3–4)	3 (3–4)	
Change from baseline	3 (2–4)	2.5 (2–3)	0.12^a^	3 (2–4)	3 (2–3)	0.40^a^
6-month follow-up	4.5 (4–5)	4 (2.25–4)		4.5 (4–5)	4 (3–5)	
Change from baseline	4 (3–4.75)	3 (2–4)	0.09^a^	4 (3–4.75)	3 (3–4)	0.40^a^
Gait speed, m/s, mean (SD)						
Discharge	0.47 (0.3)	0.30 (0.3)	0.15^a^	0.47 (0.3)	0.35 (0.3)	0.31^b^
6-month follow-up	0.67 (0.5)	0.35 (0.3)	**0.04**^a^	0.67 (0.5)	0.42 (0.3)	0.10^b^
6MWT, m, mean (SD)						
Discharge	145.8 (110.2)	80.1 (85.0)	0.08^a^	145.8 (110.2)	93.0 (84.0)	0.14^b^
6-month follow-up	211.2 (147.2)	103.0 (93.5)	**0.03**^a^	211.2 (147.2)	123.4 (90.1)	0.05^b^
Days to unassisted walking^c^	24.1 (9.7)	36.7 (16.1)	**0.03**^b^	24.1 (9.7)	35.3 (15.7)	0.05^b^

*6MWT* 6-minute walk test, *FAC* Functional Ambulation Category, *IQR* interquartile range, *SD* standard deviation

^a^Analyzed using Mann–Whitney *U* test

^b^Analyzed using independent *t*-test

^c^Exoskeleton n = 11, Usual Care (as-treated) n = 15, Usual Care (per-protocol) n = 14

Bold indicates a significant *p*-value < 0.05

## Discussion

An exoskeleton-based physical therapy program during subacute stroke rehabilitation did not result in greater improvements in walking independence by discharge when compared to standard physical therapy care. Secondary measures of walking function, physical impairment, balance, cognition, mood, and quality of life did not differ between groups at discharge or after 6 months using an intention-to-treat analysis.

Our study adds to the emerging literature surrounding the use of powered exoskeletons in stroke rehabilitation. The majority of early research has focused on chronic stroke, establishing safe usage and modest efficacy for improving gait speed [21, 50, 51]. Few randomized controlled trials have taken place in the subacute setting, and have often supplemented standard physical therapy with adjunct therapy time using an exoskeleton [52, 53]. In those studies, no differences were found between groups in gait speed, lower extremity impairment, or balance. Only one study showed greater walking independence with adjunct exoskeleton training [53]. A recently published randomized controlled study which blended exoskeleton-assisted walking with standard gait training during subacute rehabilitation, similar to our protocol, also did not find a difference between groups in improvements in the FAC, gait speed, endurance, or balance [54]. Though the specific exoskeleton device differs between emerging research, the findings across studies suggest that exoskeleton-based training is not consistently or comprehensively better than standard physical therapy at the subacute phase of stroke.

It is possible that participants in our study did not achieve a sufficient training threshold to generate large gains in walking recovery, partly attributable to the flexibility, and thus variability, of the delivered exoskeleton-based gait intervention. Participants in our study, who participated in 2.9 weekly exoskeleton sessions in place of their standard physical therapy (approximately 75% of weekly therapy), likely did not achieve the same daily walking practice as that provided in previous research of electromechanical devices exoskeleton (i.e., 5 days a week or as additional therapy) [13, 55]. Furthermore, we provided suggested training targets but allowed therapists to make their own clinical decisions; we did not strictly enforce the minimum step count during exoskeleton sessions or even after discontinuing the device. Though the mean step count per physical therapy session in the Exoskeleton group was nearly double that of the Usual Care group, 592 steps is low relative to other walking intervention studies. Klassen et al. conducted a randomized controlled trial comparing one or two daily high-dose walking-focused physical therapy sessions to standard physical therapy care during subacute stroke rehabilitation; participants receiving either higher-dose therapy regimens achieved greater 6MWT distances at follow-up than participants receiving standard care [56]. In that study, participants in standard physical therapy took an average of 580 steps per session, whereas the higher dose groups achieved 2169 and 4747 steps per session [56]. Participants in a trial conducted by Hornby et al. took 2358 steps per intervention session, and demonstrated greater gait speed and 6MWT improvements than participants in the control group [57]. Participants in our study simply may not have been sufficiently challenged.

We did not consider cardiovascular intensity in our intervention protocol, which may be another potential explanation for the lack of a training effect. Recent guidelines for stroke rehabilitation have called for more intensive therapy, suggesting that interventions for walking recovery should encompass moderate to high-intensity aerobic exercise [8, 22]. The walking interventions in the aforementioned studies by Klassen et al. and Hornby et al. were purposefully delivered at moderate to high cardiovascular intensities, which may have been a key factor in driving positive outcomes [56, 57]. Research has shown that patients participating in robotic-assisted gait training achieve only low-intensity aerobic thresholds, relative to overground walking practice [58, 59]; thus, participants in our study most likely did not achieve the moderate- to high-intensity guideline suggested for locomotor training. An important caveat of these studies showing association between aerobic intensity or stepping amount and improved walking outcomes investigated patients requiring minimal or no assistance to walk. It would have been unlikely to achieve these training intensities in our sample of non-ambulatory patients, using an exoskeleton or not, without substantial additional resources in time and staffing, though it has been accomplished in a previous study which provided ongoing support to its therapists [60].

It is important to note that the lack of significant difference from standard physical therapy does not necessitate that robotic exoskeletons should not be used in clinical practice. Indeed, our as-treated and per-protocol analyses indicate potential benefits to using an exoskeleton during physical therapy for motor function and walking (FMA-LE, gait speed, 6MWT, days to reach independence) if adherence is maintained. Furthermore, an exoskeleton is one of few options for therapists wanting to practice walking with more physically dependent patients, whether due to impairment or practical considerations (patient-to-therapist size ratio). Additionally, given that functional improvement in post-stroke walking ability is a product of neuromuscular recovery and movement compensations [61], there may have been differences between groups in the nature of walking improvement that were not captured by our included outcomes. Current research of the impact of robotic-assisted walking on other measures such as brain plasticity, muscle activation symmetry, and kinematic qualities of gait may support alternate reasons for utilizing an exoskeleton in stroke rehabilitation [51, 62, 63]. It is also important to consider the emotional and psychological benefit of standing and practicing walking for patients after stroke. This study was conducted with a nested qualitative component of patient and therapist acceptance of exoskeleton-based physical therapy; patients viewed exoskeleton-based physical therapy highly favorably and felt a sense of greater opportunity and effectiveness with exoskeleton training [64]. These findings are supported by other qualitative studies exploring therapists’ perceptions towards the utility of an exoskeleton in general practice [65, 66].

We believe a strength of our study was the pragmatic nature of the intervention protocol. Whereas many interventions during subacute stroke rehabilitation are administered and studied as an adjunct therapy, it is not always feasible to apply findings to clinical practice. Many hospitals are operating within financial and staffing constraints [67], wherein resources to administer additional therapy beyond conventional rehabilitation are not available. Our intervention protocol provided guidance as to the percentage of weekly therapy to replace with exoskeleton-based training, as well as criteria for discontinuing the exoskeleton. This flexible training protocol was informed by previous findings from robotics-assisted gait training, which have shown that ambulatory patients with stroke make greater improvements without robotics [29]. We believe a pragmatic protocol that allows flexibility for clinical decision-making is more realistic for the downstream adoption of technology-based interventions, as physical therapists often weigh expected benefits and practicality when adopting a new intervention [68]. Within this context, our trial findings offer support for the clinical use of an exoskeleton during standard inpatient physical therapy, as it is not detrimental to patient outcomes. This may be particularly relevant in treating patients with severe disability, allowing an opportunity to practice walking that would otherwise be impractical by manual facilitation alone.

Future research involving overground exoskeletons in stroke rehabilitation is warranted. By nature of design, an exoskeleton can increase the duration and repetition of walking practice while reducing therapist burden [19], and will likely become increasingly prevalent in clinical practice. Thus, it is important for future research to focus on the identification of patients for whom an exoskeleton will truly benefit. For future trials, we recommend assessing for tolerance to the training before randomization, which may even involve a trial session in the device; this may help to exclude participants who stand no chance to respond to the intervention by way of non-compliance. As highlighted earlier, other important areas for further research are the optimal targets (duration and stepping repetition) for exoskeleton-based training, as well as the level of cardiovascular intensity that can be achieved in the device by patients with subacute stroke.

### Limitations

The most obvious limitations of this study are the small sample size and loss of participants to follow-up, which increases the risk of type II errors. Due to the small sample size, we did not control for stratification or correlation of data over time in the statistical analysis, which also increases the type II error risk. Though we viewed the allowance of clinical decision making as a strength of the protocol, the lack of enforced training targets and high variability in exoskeleton-usage between participants and trainers may have played a role in the lack of significant findings. Furthermore, because we only monitored two therapy sessions per week, we did not gather a full picture of participants’ activities during physical therapy and thus it is possible that the exercise regimen for each group was more alike, or different, than observed. Using walking dependency for inclusion or exclusion from the trial may have posed another limitation, as it is rated by the assessor and allows for subjectivity depending on therapist-to-patient size differences, walking aids provided, and personal risk assessment. Despite participants being classified as non-ambulatory at enrollment, there were large ranges in physical impairment and balance which may have influenced therapists’ approach to individualized treatment and obscured a treatment response. Finally, the male-to-female ratio was greater in the intervention group, whereas the stroke population typically has an even ratio.

## Conclusions

An exoskeleton-based physical therapy program can be safely administered and integrated within inpatient stroke rehabilitation at no detriment for non-ambulatory patients with subacute stroke. This study did not show greater improvements in walking ability when comparing exoskeleton-based physical therapy to standard physical therapy. However, exploratory as-treated and per-protocol analyses showed promising findings which indicate that future research should focus on the identification of patients who will adhere to and benefit from exoskeleton training. As well, exoskeleton-based research should also focus on determining an optimal training regimen, in duration, repetition, and intensity.

## Data Availability

The dataset used during the current study are available from the corresponding author on reasonable request.

## Abbreviations

6MWT: 6 Minute Walk Test
ANCOVA: Analysis of covariance
CI: Confidence interval
FAC: Functional Ambulation Category
FMA-LE: Fugl-Meyer Assessment lower extremity component
IQR: Interquartile range
MoCA: Montreal Cognitive Assessment
SD: Standard deviation
